# Age-dependent differences in diabetes and acute hyperglycemia between men and women with ST-elevation myocardial infarction: a cohort study

**DOI:** 10.1186/1758-5996-5-34

**Published:** 2013-07-04

**Authors:** Amber M Otten, Jan Paul Ottervanger, Jorik R Timmer, Arnoud WJ van ’t Hof, Jan-Henk E Dambrink, AT Marcel Gosselink, Jan CA Hoorntje, Harry Suryapranata, Angela HEM Maas

**Affiliations:** 1 Isala klinieken, Department of Cardiology, Groot Wezenland 20, Zwolle 8011 JW, The Netherlands; 2 Department of cardiology, University Medical Center Nijmegen, Nijmegen, the Netherlands

**Keywords:** STEMI, Gender, Diabetes, Acute hyperglycemia

## Abstract

**Background:**

Both acute hyperglycemia as diabetes results in an impaired prognosis in ST-elevation myocardial infarction (STEMI) patients. It is unknown whether there is a different prevalence of diabetes and acute hyperglycemia in men and women within age-groups.

**Methods:**

Between 2004 and 2010, 4640 consecutive patients (28% women) with STEMI, were referred for primary PCI. Patients were stratified into two age groups, < 65 years (2447 patients) and ≥65 years (2193 patients). Separate analyses were performed in 3901 patients without diabetes. Diabetes was defined as known diabetes or HbA1c ≥6.5 mmol/l at admission.

**Results:**

The prevalence of diabetes was comparable between women and men in the younger age group (14% vs 12%, p = 0.52), whereas in the older age group diabetes was more prevalent in women (25% vs 17% p < 0.001). In patients without diabetes, admission glucose was comparable between both genders in younger patients (8.1 ± 2.0 mmol/l vs 8.0 ± 2.2 mmol/l p = 0.36), but in older patients admission glucose was higher in women than in men (8.7 ± 2.1 mmol/l vs 8.4 ± 2.1 mmol/l p = 0.028). After multivariable analyses, the occurrence of increased admission glucose was comparable between men and women in the younger age group (OR 1.1, 95%CI 0.9-1.5), but increased in women in the older age group (OR 1.3, 95% CI 1.1-1.7). Both diabetes and hyperglycemia were associated with a higher one-year mortality in both men and women.

**Conclusions:**

The differences between men and women in hyperglycemia and diabetes in patients with STEMI are age dependent and can only be observed in older patients. This may have implications for medical treatment and should be investigated further.

## Background

Both hyperglycemia and diabetes are independent predictors of impaired prognosis after ST elevation myocardial infarction (STEMI) [1-4]. Prevalences of both hyperglycemia and diabetes in STEMI are increased in women, which in part may explain their higher mortality rates [1,5,6]. Moreover, diabetes has been associated with a higher cardiovascular mortality in women compared to men [7-9]. In the general population however, only in elderly people diabetes is more often present in women than in men [10]. Until now, data with regard to the impact of age on the difference in prevalence of hyperglycemia and diabetes between men and women with STEMI are lacking. We investigated whether the differences in both hyperglycemia and diabetes are age-dependent within a large registry of patients with STEMI, treated with primary percutaneous coronary intervention (PCI).

## Methods

We performed an observational study including all consecutive patients admitted with STEMI, referred for primary PCI to our hospital between 2004 and 2010. Within these time frames, HbA1c and glucose were routinely measured on admission in all STEMI patients. Patients were diagnosed with STEMI if they had chest pain longer than 30 minutes and ECG changes with ST elevation greater than 2 mm in at least two precordial leads or greater than 1 mm in the limb leads. All patients were directly transported to the catheterization 4laboratory on arrival, and acute coronary angiography was performed with subsequent PCI when indicated as part of the routine treatment for all STEMI patients. The interventional strategy was at the operator’s discretion. All patients were pretreated with aspirin, heparin, and clopidogrel during transportation to the hospital according to protocol, or these drugs were administered at the emergency ward. Cardiac biomarkers were elevated in all patients. Diabetes was defined as known diabetes or a HbA1c ≥ 6.5 at admission. This HbA1c value was identified by the American Diabetes Association as diagnostic for diabetes mellitus [11]. We performed additional analysis on a group without diabetes in order to concentrate on acute hyperglycemia due to stress. We conducted a multivariate analysis with gender as a predictor of a higher than median glucose levels. We corrected for confounders based on previously described variables in the literature [1,8]. Therefore, the multivariate model consisted of gender, TIMI flow, Killip class and age.

### Data collection

Patient characteristics were registered into a dedicated database. Thrombolysis in Myocardial Infarction (TIMI) [12] flow was scored according to the TIMI flow grading system before and after PCI. Follow-up information was obtained with pre-defined time intervals of 30 days and one year using the outpatient files or by direct telephone interview by independent research nurses not involved in patient treatment., The HbA1c levels were measured on the Primus Ultra 2 affinity chromatography-HPLC (Primus Diagnostics, Kansas City, MO) with a within-run coefficient of variation of < 0.5%. The reference normal values in non diabetics were 4.0% to 6.5%. Glucose levels were measures with a Modular device (Roche Diagnostics). The reference values did not change during the study period and yearly numeric quality control data revealed that the coefficient of variation remained < 2%.

### Statistical analysis

Statistical analysis was performed using SPSS version 17.0 (SPSS Inc, Chicago, IL). Continuous data were expressed as median and inter quartile range and categorical data as percentages. In order to examinate differences in women and men, we performed the Chi^2^ test for categorical variables and one-way Anova for continuous variables. The test for significance were two-sided with an α of < 0.5%. Multivariate analyses were performed using binary logistic regression. Predictors were identified using forward, stepwise logistic regression with the likelihood ratio test of all baseline variables with an α ≥ 0.1. The three most significant values and gender were entered into the final multivariate model. Kaplan Meier was performed with the log rank test for the p-values.

## Results

A total of 4640 patients with STEMI were admitted between 2004 and 2010. Mean age of the total population was 64 ± 13 years, including 1291 women (28%). In the total population, the prevalence of hypertension was 36%, smoking 41% and hypercholesterolemia 21%. A total of 464 (10%) patients had a previous myocardial infarction.

### Effect of age

In older women, a higher killip class was observed compared to men. This difference was not present in the younger age group. The prevalence of known diabetes was 10% in men en 16% in women in the total study group (p < 0.001). Undetected diabetes was observed in 4% of men and in 5% of women (p = 0.80), resulting in a significantly different prevalence of diabetes between both genders (p < 0.001). Diabetes was associated with an increased one-year mortality in both men (OR 1.9, 1.4-2.8 95% CI) and women (OR 2.1, 1.4-3.2 95% CI).

**Figure 1:**
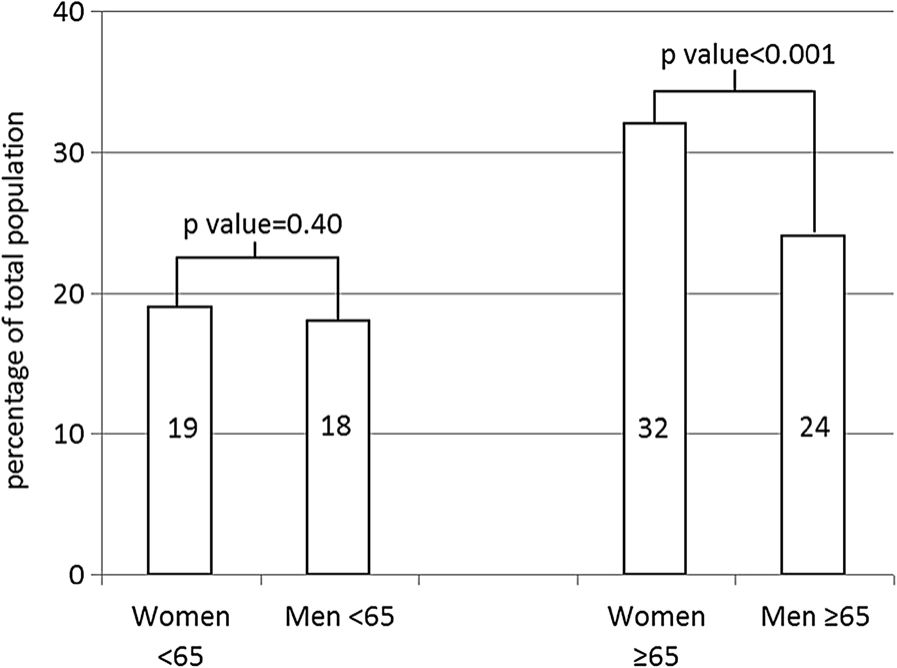
Prevalence diabetes in STEMI patients according to age and gender.

In the older age group the prevalence of diabetes was higher in women, while in the younger age group the prevalence was comparable (Table 1 and Figure 1).

**Table 1:** Prevalence of diabetes in the total study population (n = 4640) according to gender and age group

	**Women**	**Men**	**p-value**	**Women**	**Men**	**p-value**
	**<65**	**<65**		**≥65**	**≥65**	
	**N = 508**	**N = 1939**		**N = 783**	**N = 1410**	
**History of diabetes**	54(11%)	157(8%)	0.15	155(20%)	184(13%)	<0.001
Newly detected diabetes*	13(3%)	77(4%)	0.16	35(4%)	51(4%)	0.17
**Total without chronic diabetes**	439(86%)	1701(88%)	0.52	590(75%)	1171(83%)	<0.001
**Missing**	2	4		3	4	

* Patients without previously known diabetes and HbA1c ≥ 6.5% mmol/L.

### Hyperglycemia in patients without diabetes

A total of 3901 patients had no diabetes, consisting of 1029 women (26%). Of the total group, 1761 patients (45%) were aged ≥65 years. Mean admission glucose was associated with both age and gender. Mean admission glucose was 8.2 ± 2.2 mmol/l in men and 8.5 ± 2.1 in women (p = 0.001). The mean admission glucose was 8.3 ± 2.6 mmol/l in younger patients and 8.9 ± 2.6 mmol/l in older patients (p < 0.001).

Baseline characteristics of the 3901 patients, stratified to age group and gender are summarized in Table 2. Besides differences in history of hypertension and smoking there were no significant differences in risk factors between men and women in the patient group below 65 years of age. In the older patient group however, men had more often a previous history of cardiovascular disease (prior PCI, CABG, myocardial infarction or stroke) while women were more often known with hypertension.

**Table 2:** Baseline Characteristics according to gender and age groups <65 and ≥65 years in 3901 patients admitted for primary angioplasty for ST-segment elevation myocardial infarction (STEMI) without diabetes

	**Women**	**Men**	**p-value**	**Women**	**Men**	**p-value**
	**<65**	**<65**		**≥65**	**≥65**	
	**N = 439**	**N = 1701**		**N = 590**	**N = 1171**	
**Age (year)**	55(48–60)	54(49–59)	0.41	75(70–81)	73(69–78)	<0.001
**History of, n (%)**						
**MI**	19(4%)	113(7%)	0.07	39(7%)	176(15%)	<0.001
**CABG**	5(1%)	30(2%)	0.36	13(2%)	67(6%)	0.001
**PCI**	21(5%)	117(7%)	0.11	36(6%)	141(12%)	<0.001
**Stroke**	5(1%)	21(1%)	0.87	17(3%)	57(5%)	0.05
**Risk factors**						
**History of hypertension**	142(32%)	431(26%)	0.004	267(45%)	432(37%)	0.001
**Positive family history**	227(53%)	841(51%)	0.43	170(30%)	310(27%)	0.34
**Current smoking**	280(65%)	963(57%)	0.004	123(21%)	263(23%)	0.41
**Hypercholesterolemia**	65(15%)	320(19%)	0.06	99(17%)	205(18%)	0.66
**Admission data**						
**Glucose (mmol/l)**	8.2 ± 2.2	8.0 ± 2.2	0.36	8.7 ± 2.1	8.4 ± 2.1	0.03
**Killip class = 1**	412(94%)	1619(96%)	0.22	519(88%)	1066(91%)	0.05
**TIMI-3 before PCI**	105(27%)	335(22%)	0.06	116(24%)	219(22%)	0.36

Continuous variables are displayed as median and interquartile range.

Glucose level at admission was comparable between men and women in the younger age group, but higher in women at older age (Table 2) Also, the prevalence of admission glucose above the median was comparable within both genders at younger age in the multivariate model (OR 1.1, 0.9-1.5 95% CI) while in the older age group, admission glucose above the median was more frequent in women. (OR 1.3, 1.1-1.7 95% CI) This age-related difference remained after multivariate analyses.

In patients without diabetes, acute hyperglycemia was associated with an increased one-year mortality in both men (OR 2.2, 1.5-3.4 95% CI) and women (OR 2.9, 1.6-5.4 95% CI).

**Figure 2:**
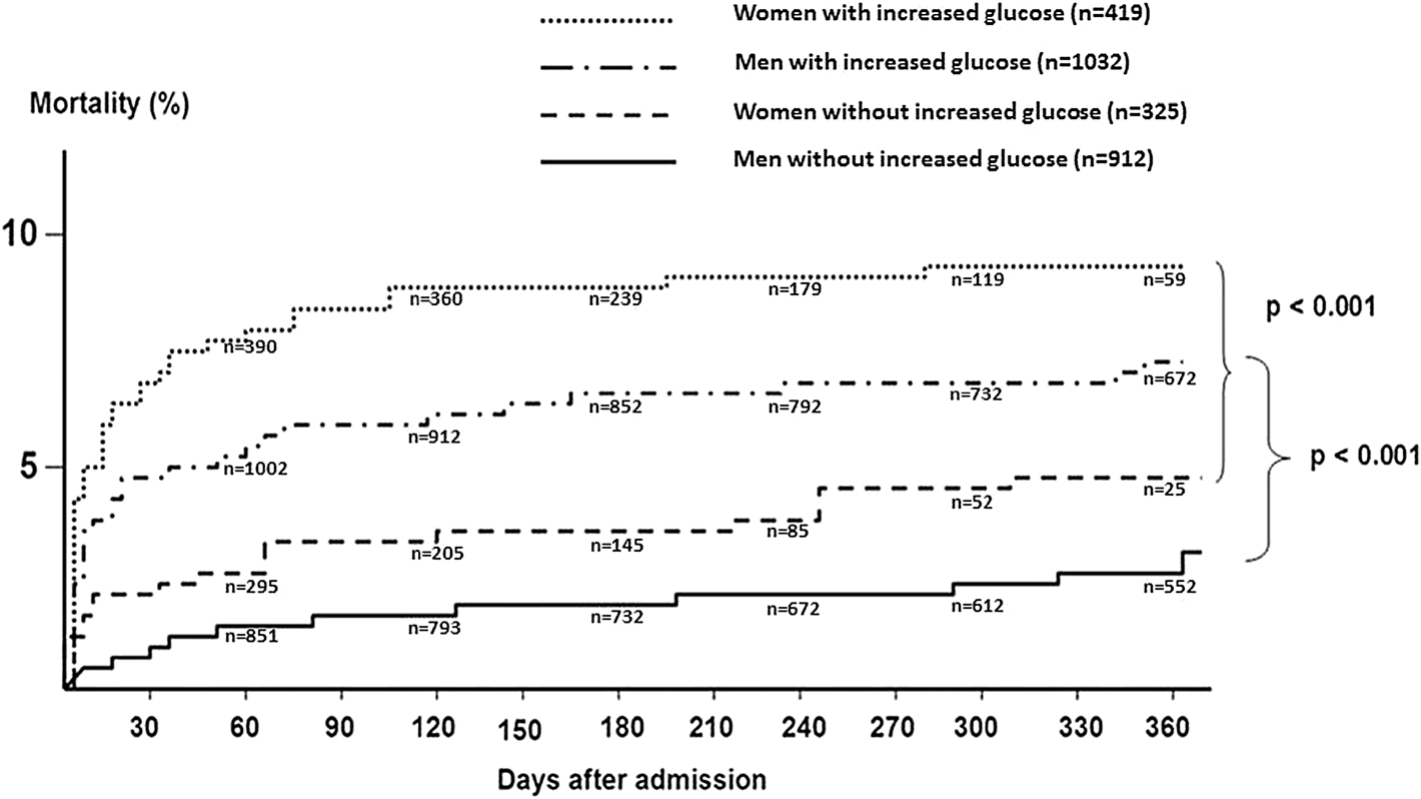
Kaplan Meier mortality curves for patients without a history of diabetes, dichotomized into higher and lower than median glucose and stratified to gender (n = 3901).

Mortality curves for patients without a history of diabetes are dichotomized into higher and lower than median glucose in women (8.1 mmol/l) and men (7.8 mmol/l) are depicted in Figure 2.

## Discussion

In our present study of patients treated with primary PCI for STEMI the prevalence of diabetes was higher in older women compared to similarly aged men. In younger patients, however, we found no differences between men and women. Also, in patients without diabetes, a higher admission glucose could only be demonstrated in older women as compared to older men. Both diabetes and increased admission glucose in patients without diabetes, were associated with a higher one-year mortality in both women and men.

Our study confirmed the increased prevalence of diabetes and acute hyperglycemia in women compared to men [5-7]. A new finding however in STEMI patients is that this association is age-dependent and only present in the older age-group. Diabetes (both known and unknown) confers to a greater risk for adverse cardiovascular events in women than in men [6,7]. Therefore, the increased risk induced by diabetes in patients presenting with STEMI is predominantly observed in older women.

In assessing the risk of adverse events in patients presenting with STEMI, both age and gender are important factors to consider. Importantly, the increased prevalence of diabetes in older women compared to older man is part of a different risk profile. Consistent with the literature, we found that men more frequently had ischemic heart disease in the medical history [6,7]. Hypertension however was more common in both older and younger women, and this is interesting because hypertension has been associated with the development of diabetes [13-15]. Hypertension may be an early sign of microvascular disease and increased risk of pre-diabetes and STEMI in (aging) women. The association between hypertension and diabetes is also important, since both risk factors induce microvascular and more diffuse coronary artery disease, which is more prevalent in older women [16-19].

Our findings may have implications for medical treatment for patients with STEMI, since it has been shown that some antiplatelet drugs are more effective in patients with diabetes [20,21]. Particularly since older women with abnormal glucose metabolism have a worse prognosis, optimal medical treatment is mandatory in this subgroup.

It is important to discriminate diabetes from acute hyperglycemia at admission [1,22]. High admission glucose in patients with diabetes is mainly due to glucose intolerance in the setting of diabetes. Whereas in patients without diabetes, hyperglycemia is probably associated with acute stress, induced by abnormal hemodynamics [1,23,24].

There are several explanations for the increased prevalence of acute hyperglycemia in older women without diabetes. Firstly, although we excluded patients with increased HbA1c, several older women may have had abnormal chronic glucose metabolism. Therefore, women are more susceptible for hyperglycemia in response to a stressor, as compared to patients with completely normal glucose metabolism. Secondly, older women with STEMI may have had more acute stress as there is evidence that women more often present with cardiogenic shock compared to men [25]. Also, in our study population older women had more often signs of heart failure on admission as compared to older men, whereas in the younger age group there was no difference in heart failure between men and women. However, after adjustment for the observed differences in heart failure, we found that older women still had increased admission glucose levels. Finally, gender-differences to stress in STEMI patients may be more present in elderly woman than in similarly aged men.

Our study has several limitations. The number of patients in some subgroups were small, and the study was not powered to detect small differences between these subgroups. Also, the sample size was too small to demonstrate survival differences between men and women within the different age groups. Information regarding renal failure, liver failure, obesity, physical activity, inflammatory markers and socioeconomic status were lacking. Therefore, we were unable to adjust for these potential confounders. Finally, our data cannot be extrapolated to non-STEMI patients,non-cardiac patients admitted to intensive care wards, or unstable patients since our study included only STEMI patients and only 8% of these patients had a killip class higher than 1.

## Conclusion

In STEMI, diabetes and hyperglycemia on admission is more prevalent in older women compared to similarly aged men. This association was not prevalent in younger patients. We observed an independent increased risk of acute hyperglycemia in older women without diabetes and therefore, older women may have an increased stress response. Both acute hyperglycemia and diabetes are associated with a worse prognosis in both women and men. More research is needed to elucidate these age-dependent gender differences and to explore whether tailored treatment can improve prognosis.

## Abbreviations

LDH: Lactaatdehydrogenase; NSTEMI: Non ST Elevation Myocardial Infarction; PPCI: Primary Percutaneous Coronary Intervention; STEMI: ST Elevation Myocardial Infarction; TIMI: Thrombolysis In Myocardial Infarction.

## Competing interest

The authors declare that they have no competing interest.

## Authors’ contributions

AO participated in the design of the study, drafting of the manuscript, analysis and interpretation of the data. JPO participated in the design of the study, data collection drafting of the manuscript and interpretation of the data. JT participation in design of the study and interpretation of the data. AH participated in data collection and revised the manuscript. JD participated in data collection and revised the manuscript MG participated in data collection and revised the manuscript. JH participated in data collection and revised the manuscript. HS participated in data collection and revised the manuscript. AM participated in the design of the study and drafting of the manuscript. All authors read and approved the final manuscript.

## Authors' information

Amber M Otten, Jan Paul Ottervanger, Jorik R Timmer, Arnoud WJ van ’t Hof, Jan-Henk E Dambrink, AT Marcel Gosselink, Jan CA Hoorntje, Harry Suryapranata, on behalf of the Zwolle Myocardial Infarction Study Group.

## References

[B1] Timmer JR Hoekstra M Nijsten MWN Van Der Horst IC Ottervanger JP Slingerland RJ Dambrink JHE Bilo HJ Zijlstra F Van T Hof AWJ Prognostic value of admissio glycosylated hemoglobin and glucose in nondiabetic patients with st-segment elevation myocartial infarction treates with percutaneous coronary intervention Circulation 2011 124 704 711 10.1161/CIRCULATIONAHA.110.985911 21768543

[B2] Isomaa B Almgren P Tuomi T Forsén B Lahti K Nissén M Taskinen MR Groop L Cardiovascular morbidity and mortality associated with the metabolic syndrome Diabetes Care 2001 24 683 689 10.2337/diacare.24.4.683 11315831

[B3] Thalib L Zubaid M Rashed W Suwaidi JA Almahmeed W Alozairi E Alanbaei M Sulaiman K Amin H Al-Motarreb A Impact of diabetic status on the hyperglycemia-induced adverse risk of short term outcomes in hospitalized patients with acute coronary syndromes in the Middle East: Findings from the Gulf registry of Acute Coronary Events (Gulf RACE) Clin Med Res 2011 9 32 37 10.3121/cmr.2010.946 20852085 PMC3064757

[B4] Monteiro S Monteiro P Gonçalves F Freitas M Providěncia LA Hyperglycaemia at admission in acute coronary syndrome patients: Prognostic value in diabetics and non-diabetics Eur J Cardiovasc Prev Rehabil 2010 17 155 159 10.1097/HJR.0b013e32832e19a3 20110816

[B5] Berger JS Elliott L Gallup D Roe M Granger CB Armstrong PW Simes RJ White HD Van de Werf F Topol EJ Hochman JS Newby LK Harrington RA Califf RM Becker RC Douglas PS Sex differences in mortality following acute coronary syndromes JAMA 2009 302 874 882 10.1001/jama.2009.1227 19706861 PMC2778841

[B6] Hailer B Naber C Koslowski B Van Leeuwen P Schäfer H Budde T Jacksch R Sabin G Erbel R Myocardial Infarction Network Essen Gender-related differences in patients with ST-elevation myocardial infarction: Results from the registry study of the ST elevation myocardial infarction network Essen Clin Cardiol 2011 34 294 301 10.1002/clc.20916 21557255 PMC6652718

[B7] Hu G Jousilahti P Qiao Q Peltonen M Katoh S Tuomilehto J The gender-specific impact of diabetes and myocardial infarction at baseline and during follow-up on mortality from all causes and coronary heart disease J Am Coll Cardiol 2005 45 1413 1418 10.1016/j.jacc.2005.01.039 15862411

[B8] Natarajan S Liao Y Cao G Lipsitz SR McGee DL Sex differences in risk for coronary heart disease mortality associated with diabetes and established coronary heart disease Arch Intern Med 2003 163 1735 1740 10.1001/archinte.163.14.1735 12885690

[B9] Huxley R Barzi F Woodward M Excess risk of fatal coronary heart disease associated with diabetes in men and women: meta-analysis of 37 prospective cohort studies BMJ 2006 332 73 78 10.1136/bmj.38678.389583.7C 16371403 PMC1326926

[B10] The Decode Study Group Age- and sex-specific prevalences of diabetes and impaired glucose regulation in 13 European cohorts Diabetes Care 2003 26 61 69 10.2337/diacare.26.1.61 12502659

[B11] American Diabetes Association Diagnosis and classification of diabetes mellitus Diabetes Care 2012 35 S64 S71 22187472 10.2337/dc12-s064 PMC3632174

[B12] The TIMI study group The Thrombolysis in Myocardial Infarction (TIMI) trial: Phase 1 findings N Engl J Med 1985 312 932 936 4038784 10.1056/NEJM198504043121437

[B13] Mozaffarian D Marfisi R Levantesi G Silletta MG Tavazzi L Tognoni G Valagussa F Marchioli R Incidence of new-onset diabetes and impaired fasting glucose in patients with recent myocardial infarction and the effect of clinical and lifestyle factors Lancet 2007 370 667 675 10.1016/S0140-6736(07)61343-9 17720018

[B14] Conen D Ridker PM Mora S Buring JE Glynn RJ Blood pressure and risk of developing type 2 diabetes mellitus: the Women's Health Study Eur Heart J 2007 28 2937 2943 10.1093/eurheartj/ehm400 17925342

[B15] Gress TW Nieto J Shahar E Wofford MR Brancati FL Hypertension and antihypertensive therapy as risk factors for type 2 diabetes mellitus N Engl J Med 2000 43 905 912 10738048 10.1056/NEJM200003303421301

[B16] Bairey Merz CN Kelsey SF Pepine CJ Reichek N Reis SE Rogers WJ Sharaf BL Sopko G The Women’s Ischemia Syndrome Evaluation (WISE) study: Protocol design, methodology and feasibility report J Am Coll Cardiol 1999 33 1453 1461 10.1016/S0735-1097(99)00082-0 10334408

[B17] Reis SE Holubkov R Lee JS Sharaf B Reichek N Rogers WJ Walsh EG Fuisz AR Kerensky R Detre KM Sopko G Pepine CJ Coronary flow velocity response to adenosine characterizes coronary microvascular function in women with chest pain and no obstructive coronary disease: Results from the pilot phase of the Women’s Ischemia Syndrome Evaluation (WISE) study J Am Coll Cardiol 1999 33 1469 1475 10.1016/S0735-1097(99)00072-8 10334410

[B18] Wessel TR Arant CB McGorray SP Sharaf BL Reis SE Kerensky RA von Mering GO Smith KM Pauly DF Handberg EM Mankad S Olson MB Johnson BD Merz CN Sopko G Pepine CJ Coronary microvascular reactivity is only partially predicted by atherosclerosis risk factors or coronary artery disease in women evaluated for suspected ischemia: Results from the NHLBI Women's Ischemia Syndrome Evaluation (WISE) Clin Cardiol 2007 30 69 74 10.1002/clc.19 17326061 PMC6653045

[B19] Waller BF Palumbo PJ Lie JT Roberts WC Status of the coronary arteries at necropsy in diabetes mellitus with onset after age 30 years: Analysis of 229 diabetic patients with and without clinical evidence of coronary heart disease and comparison to 183 control subjects Am J Med 1980 69 498 506 10.1016/S0149-2918(05)80002-5 7424939

[B20] Wiviott SD Braunwald E Angiolillo AJ Meisel S Dalby AJ Verheugt FW Goodman SG Corbalan R Purdy DA Murphy SA McCabe CH Antman EM EM; TRITON-TIMI 38 Investigators Greater clinical benefit of more intensive oral antiplatelet therapy with prasugrel in patients with diabetes mellitus in the trial to assess improvement in therapeutic outcomes by optimizing platelet inhibition with prasugrel − Thrombolysis in Myocardial Infarction 38 Circulation 2008 118 1626 1636 10.1161/CIRCULATIONAHA.108.791061 18757948

[B21] Ferreiro JL Angiolillo AJ Diabetes and antiplatelet therapy in acute coronary syndrome Circulation 2011 123 798 813 10.1161/CIRCULATIONAHA.109.913376 21343595

[B22] Anatharaman R Heatley M Weston CF Hyperglycemia in acute coronary syndromes: risk-marker or therapeutic target? Heart 2009 95 697 703 10.1136/hrt.2008.146027 18697807

[B23] Capes SE Hunt D Malmberg K Gerstein HC Stress hyperglycaemia and increased risk of death after myocardial infarction in patients with and without diabetes: a systematic overview Lancet 2000 355 773 778 10.1016/S0140-6736(99)08415-9 10711923

[B24] Norhammar A Tenerz A Nilsson G Hamsten A Efendíc S Rydén L Malmberg K Glucose metabolism in patients with acute myocardial infarction and no previous diagnosis of diabetes mellitus: a prospective study Lancet 2002 359 2140 2144 10.1016/S0140-6736(02)09089-X 12090978

[B25] Koeth O Zahn R Heer T Bauer T Juenger C Klein B Gitt AK Senges J Zeymer U Gender differences in patients with acute ST-elevation myocardial infarction complicated by cardiogenic shock Clin Res Cardiol 2009 98 781 786 10.1007/s00392-009-0080-7 19856196

